# Eosinophilic inflammation: a key player in COPD pathogenesis and progression

**DOI:** 10.1080/07853890.2024.2408466

**Published:** 2024-10-07

**Authors:** Yueh-Lun Lee, Didik Setyo Heriyanto, Fara Silvia Yuliani, Vincent Laiman, Lina Choridah, Kang-Yun Lee, Jer-Hwa Chang, Kian Fan Chung, Li-Te Chang, Ta-Yuan Chang, Xiao-Yue Chen, Syue-Wei Peng, Kai-Jen Chuang, Hsiao-Chi Chuang

**Affiliations:** a Department of Microbiology and Immunology, School of Medicine, College of Medicine, Taipei Medical University, Taipei, Taiwan; b Department of Anatomical Pathology, Faculty of Medicine, Public Health, and Nursing, Universitas Gadjah Mada – Dr. Sardjito Hospital, Yogyakarta, Indonesia; c Department of Pharmacology and Therapy, Faculty of Medicine, Public Health and Nursing, Universitas Gadjah Mada, Yogyakarta, Indonesia; d Department of Radiology, Faculty of Medicine, Public Health, and Nursing, Universitas Gadjah Mada – Dr. Sardjito Hospital, Yogyakarta, Indonesia; e Division of Pulmonary Medicine, Department of Internal Medicine, Shuang Ho Hospital, Taipei Medical University, New Taipei City, Taiwan; f Division of Pulmonary Medicine, Department of Internal Medicine, School of Medicine, College of Medicine, Taipei Medical University, Taipei, Taiwan; g School of Respiratory Therapy, College of Medicine, Taipei Medical University, Taipei, Taiwan; h Division of Pulmonary Medicine, Department of Internal Medicine, Wan Fang Hospital, Taipei Medical University, Taipei, Taiwan; i National Heart and Lung Institute, Imperial College London, London, UK; j Department of Environmental Engineering and Science, Feng Chia University, Taichung, Taiwan; k Department of Occupational Safety and Health, College of Public Health, China Medical University, Taichung, Taiwan; l School of Public Health, College of Public Health, Taipei Medical University, Taipei, Taiwan; m Department of Public Health, School of Medicine, College of Medicine, Taipei Medical University, Taipei, Taiwan; n Cell Physiology and Molecular Image Research Center, Wan Fang Hospital, Taipei Medical University, Taipei, Taiwan

**Keywords:** Airway remodeling, asthma, emphysema, eosinophilia, precision medicine

## Abstract

**Background:**

Chronic Obstructive Pulmonary Disease (COPD) remains a significant public health challenge due to its high morbidity and mortality rates. Emerging research has identified eosinophilic inflammation as a crucial factor in the pathogenesis and exacerbation of COPD, warranting a detailed exploration of its underlying mechanisms and therapeutic implications.

**Objective:**

This review aims to elucidate the role of eosinophils in COPD, focusing on their contribution to airway remodeling, exacerbation frequency, and the inflammatory cascade.

**Methods:**

We conducted a comprehensive literature review of recent studies that discuss the pathophysiological role of eosinophils in COPD and the clinical outcomes associated with modulating eosinophilic activity.

**Results:**

Eosinophils contribute to COPD progression by releasing cytotoxic proteins and cytokines that intensify the inflammatory response and airway alterations. Targeting specific eosinophil-related cytokines with monoclonal antibodies or receptor antagonists may potentially reduce eosinophil counts, mitigate exacerbations, and improve patient outcomes.

**Conclusion:**

Understanding eosinophilic involvement in COPD can facilitate the development of precision medicine approaches, offering more tailored and effective treatment options. Future research should continue to focus on the integration of eosinophil biomarkers in clinical practice to enhance therapeutic decisions and management strategies for COPD patients.

## POINTS FOR CLINICAL PRACTICE

1. Elevated eosinophil levels in COPD may be used to tailor treatment plans.
2. Biologic therapies targeting eosinophils could be applied to manage eosinophilic COPD severity.
3. Personalized treatment strategies based on eosinophil levels can enhance COPD management.
4. Regular monitoring of eosinophil levels in COPD patients is crucial as it may influences treatment responses and disease progression.
5. A deeper understanding of the role of eosinophils in COPD pathophysiology helps in making informed therapeutic decisions and developing targeted therapies.

## 1. Introduction

Chronic obstructive pulmonary disease (COPD), a prevalent and debilitating condition, significantly undermines global health by causing persistent respiratory symptoms and airflow limitation due to airway and/or alveolar abnormalities, often to the result of significant exposure to noxious particles or gases. According to the World Health Organization, COPD led to 3.23 million deaths in 2019, ranking as the third primary cause of death worldwide [1]. The considerable prevalence of COPD, alongside its impact on mortality and disability, underscores the urgent need for effective preventive and therapeutic strategies.

Central to the evolution and advancement of COPD is inflammation [2]. Persistent exposure to irritants, notably tobacco smoke, triggers chronic inflammatory responses in the airways and lungs, typically marked by an increase in neutrophils, macrophages, and T lymphocytes in the airway walls [2]. These changes lead to the narrowing and obstruction of the small airways, contributing to airflow limitation. However, the inflammatory landscape in COPD is heterogeneous, with significant variability among patients, suggesting diverse inflammatory phenotypes.

Eosinophilic inflammation represents one such phenotype, characterized by the involvement of eosinophils, a type of white blood cell integral to the body’s immune defense, particularly against parasitic infections [3]. However, they also play a role in the pathogenesis of various inflammatory conditions, including asthma and COPD [4]. In some individuals with COPD, eosinophils contribute significantly to airway inflammation, a condition termed eosinophilic COPD [2]. This phenotype is associated with a greater frequency of exacerbations and a distinct response to treatment compared to the non-eosinophilic form of COPD [2]. Beyond eosinophilic inflammation, other elements like chitotriosidase, neutrophilic inflammation, tissue remodeling, and virus-induced dysfunction of airway immunity also play crucial roles in COPD’s development, complicating its management further.

A wide variation in eosinophilic COPD prevalence has been reported, spanning from 9.58% to 66.88%, with an average prevalence of 54.95% [5]. When comparing eosinophilic and non-eosinophilic COPD groups, the male to female ratios are distinct, highlighting a disparity. Traditionally, eosinophilic airway inflammation is identified through sputum eosinophil counts, but peripheral blood eosinophil counts are emerging as a potential diagnostic tool for defining eosinophilic COPD phenotypes [6]. This approach, particularly using blood eosinophil counts of ≥2% or ≥150 cells/µL, is linked to increased exacerbations and a better response to specific treatments in certain COPD cases [7]. Despite these insights, the impact of eosinophilic versus non-eosinophilic phenotypes on healthcare resource use and costs remains unclear, especially within the eosinophilic COPD category.

This review delves into eosinophilic inflammation’s role in COPD’s onset and progression, providing a detailed exploration of current knowledge on this topic, including the basic biology of eosinophils, mechanisms of eosinophilic inflammation, its impact on COPD, and therapeutic strategies targeting this inflammation. Additionally, the review will discuss risk factors for eosinophilic inflammation in COPD, offering a comprehensive understanding of this critical aspect of COPD pathophysiology.

## 2. Methods

### 2.1. Literature search strategy

To conduct a comprehensive and systematic literature review, we searched three major databases: PubMed, Web of Science, and Google Scholar. Our search targeted literature published from January 1, 2020, to April 25, 2024, ensuring the inclusion of the most current research available on the topic. The search employed a combination of keywords specifically related to the role of eosinophils in COPD. These keywords included: ‘eosinophil’, ‘eosinophilic inflammation’, ‘eosinophilia’, ‘COPD’, ‘airway remodeling’, ‘bronchitis’, ‘emphysema’, ‘exacerbation’, ‘biomarker’, and ‘therapeutic strategies’. Using these keywords in various combinations helped ensure an exhaustive search of relevant literature.

### 2.2. Study selection

The initial search yielded a pool of articles, which were first screened based on their titles and abstracts to determine relevance to the topic of eosinophilic inflammation in COPD. These selected articles then underwent a full-text review to confirm their relevance and assess their quality. Both original research papers, review articles and conference papers were included. There were no geographic restrictions, but papers needed to be published in English to ensure comprehensibility and applicability in broader contexts.

## 3. Eosinophils within the immune framework

Eosinophils, a specialized subgroup of white blood cells, are pivotal in the body’s defense mechanisms, particularly against parasitic invasions and in allergic responses [8]. Originating in the bone marrow, these cells traverse the bloodstream and migrate to sites of infection or inflammation upon encountering pathogens or allergens, releasing a variety of substances to neutralize or eradicate the threat [9]. In an inflammatory context, eosinophils amplify the inflammatory process by discharging cytokines and chemokines, which recruit additional immune cells to the inflammation site, and by secreting toxic substances that cause tissue damage [2].

Eosinophils straddle the line between innate and adaptive immunity, contributing to the body’s immediate defense and the more specialized, later response to specific pathogens. They act as phagocytes, engulfing microbes, and interact with T cells and B cells, aiding in a coordinated immune response [10]. Furthermore, eosinophils help regulate the immune response, either amplifying or suppressing the activities of other immune cells, thus playing a critical role in maintaining immune homeostasis [11]. Besides their immunological roles, eosinophils are involved in tissue repair and metabolic regulation, highlighting their multifunctionality within the body [3]. However, dysregulated eosinophil activity can contribute to various diseases, emphasizing the importance of understanding eosinophil biology in health and disease.

## 4. Mechanisms underpinning eosinophilic inflammation

**Figure 1.**
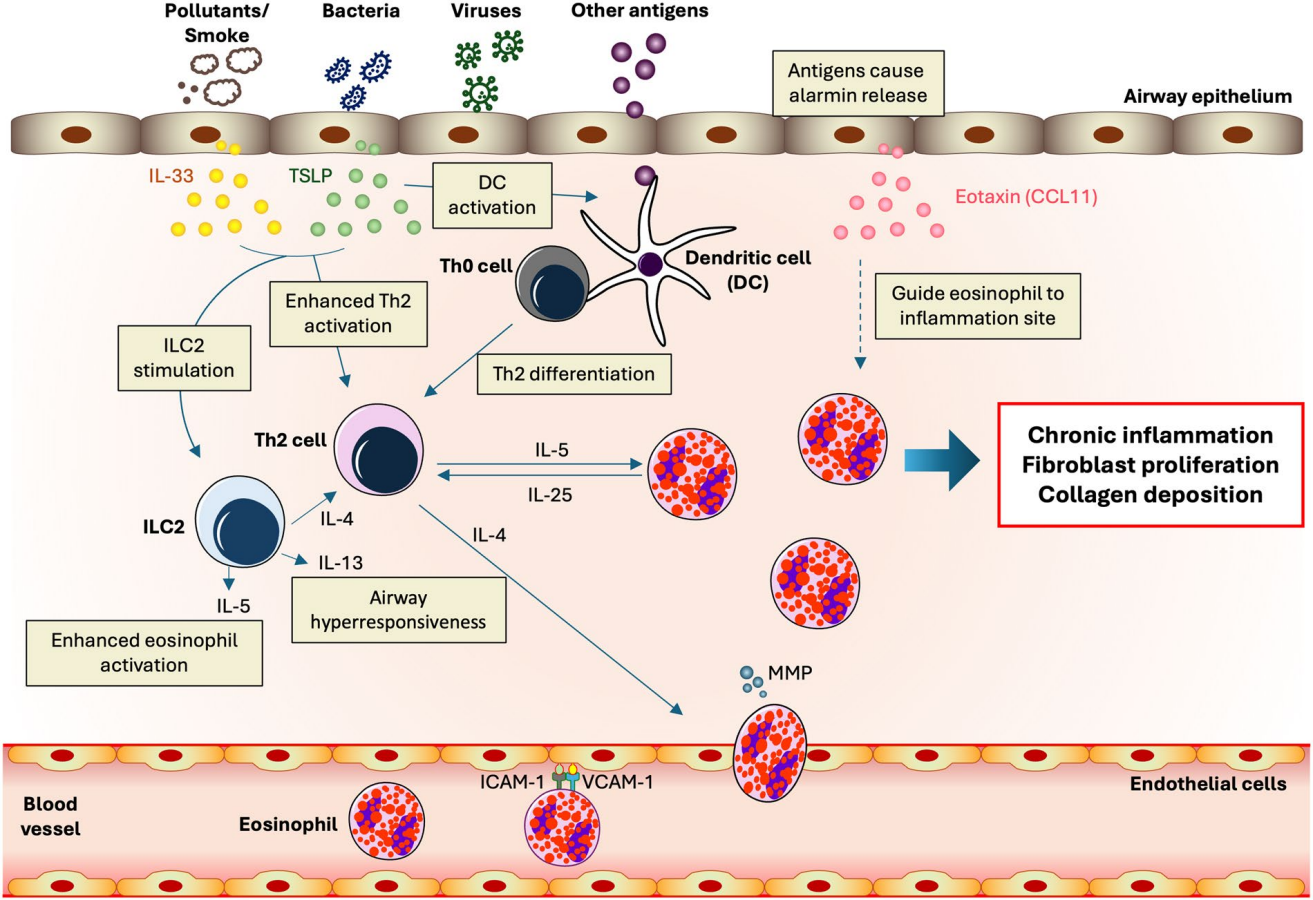
Mechanism of Antigen-Induced alarmin release and subsequent immune response leading to airway hyperresponsiveness. Illustration of antigens inducing the release of alarmins including thymic stromal lymphopoietin (TSLP), eotaxin, and interleukin-33 (IL-33). The antigens are detected by dendritic cells, which in turn activate T-helper type 2 (Th2) cells and innate lymphoid cells (ILC2). The Th2 cells release cytokines, including IL-4, which promotes the infiltration of eosinophils. These eosinophils bind to vascular cell adhesion molecule-1 (VCAM-1) and intercellular adhesion molecule-1 (ICAM-1) on endothelial cells, facilitating their migration to the site of inflammation guided by eotaxin. This process leads to airway hyperresponsiveness, characterized by inflammation, fibrosis, and collagen deposition.

This complex immunological response, marked by eosinophil accumulation and activation in tissues, often arises from allergic reactions, parasitic infections, or specific diseases (Figure 1). It commences with an initial trigger that activates immune responses, with T helper 2 (Th2) cells playing a crucial role by producing cytokines that drive eosinophil growth, differentiation, and survival [12]. The antigens induced the release of alarmins including thymic stromal lymphopoietin (TSLP), eotaxin, and interleukin-33 (IL-33) [4,13]. The antigens are detected by dendritic cells, which, along with TSLP, activate Th2 cells and innate lymphoid cells (ILC2). The Th2-derived IL-4 induces the expression of adhesion molecules (VCAM-1 and ICAM-1) on endothelial cells, facilitating eosinophil binding to blood vessel linings [4, 13]. It also enhances integrins on eosinophils, promoting their interaction with these adhesion molecules. Furthermore, IL-4 stimulates the secretion of chemokines, particularly eotaxins such as CCL11, CCL24, and CCL26, creating a chemotactic gradient that directs eosinophil migration toward sites of inflammation. The interaction between these chemokines and their receptor, CCR3, on eosinophils activates signaling pathways that promote their motility [4]. IL-4 increases endothelial cell permeability, aiding eosinophil transmigration, and modulates matrix metalloproteinases (MMPs) to degrade the extracellular matrix, facilitating tissue entry. Eosinophils, upon recruitment to the inflammation site, become activated and release mediators that cause tissue damage and perpetuate inflammation. They also produce cytokines and chemokines, contributing to a complex network of cell interactions that sustain the inflammatory state. In eosinophilic inflammation, cytokines and chemokines play a central role by first activating Th2 cells, which then produce key cytokines such as IL-4, IL-5, and IL-13 [14]. The cytokines IL-4 and IL-13 are essential for prompting B cells to produce IgE and IL-5 involved in the development and survival of eosinophils [15]. IL-5 promotes the differentiation and activation of eosinophils in the bone marrow, which is crucial for combating infections and managing allergic responses [16,17]. Eotaxin and RANTES guide eosinophils to the inflammation site through chemotactic signals, where IL-5 further enhances their activation and longevity [15,16]. This process creates a self-sustaining loop, as eosinophils emit additional cytokines that perpetuate the Th2-dominated response and engage with other cell types like endothelial cells and fibroblasts to intensify the inflammatory state [8]. Meanwhile, the mediators released by activated eosinophils, such as major basic protein, contribute to tissue damage and the characteristic symptoms of eosinophilic disorders, including airway hyperreactivity and excess mucus production [18]. This sequence highlights the dual role of cytokines and chemokines in both initiating and exacerbating inflammatory responses in eosinophilic conditions. The regulation of eosinophil activity involves multiple signaling pathways, including JAK-STAT, PI3K, and MAPK, which govern eosinophil proliferation, survival, and function [19,20].

Chronic eosinophilic inflammation can lead to tissue remodeling and fibrosis, with eosinophil mediators and cytokines stimulating fibroblast proliferation and collagen deposition, resulting in permanent tissue damage [21,22]. During chronic eosinophilic inflammation, activated eosinophils release various mediators like major basic protein, eosinophil cationic protein, and eosinophil-derived neurotoxin, which directly contribute to ongoing tissue damage and inflammation [3]. Upon activation, eosinophils secrete a range of cytokines, including IL-1β, IL-4, IL-5, IL-13, TNF-α, and TGF-β [23]. These cytokines are integral to the regulation of IgE synthesis and inflammation, contributing to airway hyperresponsiveness, mucus production, and fibrosis. For instance, IL-5 is crucial in the recruitment of eosinophils to sites of inflammation [24,25]. Eosinophil activation in response to pharmacological, hormonal, infectious, or environmental stimuli initiates a sustained, potentially IL-5-dominated, Th2-driven immune response [24,25]. This response impacts the lungs, leading to airway hyperresponsiveness and chronic remodeling. This forms a feedback loop that can intensify and perpetuate inflammatory responses. IL-5 not only stimulates eosinophil activation and survival but also boosts their capacity to produce more IL-5 and other pro-inflammatory cytokines [25]. This creates an autocrine loop in which eosinophils maintain and escalate their own activity, resulting in persistent and chronic inflammation. Additionally, the IL-13 was reported to promote the release of TGFβ through IL-13 receptor. The IL-13Rα2, a receptor of IL-13, was reported to function as a signaling receptor, as evidenced by its high-affinity binding to IL-13 [26]. The IL-13 can then promote the recruitment and activation of eosinophils, which are significant sources of TGF-β. This interaction between IL-13Rα2 and IL-13 can induce the activation of the TGFβ1 promoter and mediate the release of TGF-β [26]. Given these roles, IL-13Rα2 emerges as a potential target for treating TH2-mediated inflammation, which is characterized by high-level expression of surface IL-13Rα2 on effector cells. These TGF-β, a significant driver of fibrosis, stimulates fibroblasts, the cells responsible for collagen and extracellular matrix production. As fibroblasts respond to these signals, they proliferate and significantly increase collagen output, leading to excessive collagen deposition. This process thickens and stiffens tissues, a condition known as fibrosis, which gradually alters the tissue’s normal architecture and function [27]. Such changes are particularly detrimental in organs like the lungs, where increased fibrosis can reduce elasticity, impair lung function, and severely restrict breathing, as seen in severe chronic respiratory conditions [28,29]. To counteract this, the body employs mechanisms including regulatory T cells (Tregs) and anti-inflammatory cytokines such as IL-10 and TGF-β, which work to limit eosinophilic inflammation and mitigate potential tissue damage, highlighting the complex interplay of immune regulation in maintaining tissue integrity [30,31].

## 5. Eosinophilic inflammation’s role in COPD

Eosinophilic inflammation may contribute to the COPD severity, offering a detailed perspective on a disease traditionally associated with neutrophil-driven inflammation. Clinically, a specific group of COPD patients shows signs of eosinophilic inflammation, highlighting the disease’s complex and varied nature [32]. This inflammation is linked with Th2 mediators in the airways, similar to those seen in asthma, emphasizing the diverse inflammatory profiles that can influence treatment and outcomes. Also, eosinophilic inflammation can contribute to the development and exacerbation of COPD through several mechanisms that ultimately result in airway remodeling, decline in lung function, and increased exacerbation symptoms [25, 33,34].

Eosinophils are particularly impactful in COPD because they release inflammatory mediators such as eosinophil cationic protein, major basic protein, and eosinophil-derived neurotoxin [35]. These substances contribute to inflammation, airway remodeling, and hyperresponsiveness—all central features of COPD [36]. Additionally, eosinophils are involved in excessive mucus production, exacerbating the obstruction in the airways that is characteristic of COPD [13]. The molecular basis of eosinophilic inflammation in COPD, including specific gene and cytokine expression, is essential for developing targeted treatments. Central to this process are cytokines such as IL-5, which is crucial for the survival and activation of eosinophils and IL-13, which contributes to airway hyperreactivity and mucus production [17]. Additionally, Eotaxin-1 specifically recruits eosinophils to the airways by interacting with the CCR3 receptor [37]. Alongside these cytokines, IL-33 and Thymic stromal lymphopoietin (TSLP) further amplify Th2 cell responses, thereby exacerbating eosinophilic activity [38]. The molecular signaling pathways involved, particularly the Th2 pathway and JAK-STAT signaling mechanisms, facilitate the transcription and action of these cytokines [19].

In cases of COPD linked with eosinophilic inflammation, various mechanisms lead to detrimental changes in the structure of the airways. Eosinophils release compounds that encourage the growth of fibroblasts, significantly driven by cytokine Transforming TGF-β, which leads to fibrosis and an increase in collagen within the airway walls [39]. This buildup of collagen reduces the diameter of the airways, restricting airflow and increasing resistance to breathing. Moreover, substances from eosinophils also cause an increase in the size and number of smooth muscle cells in the airway wall, increase airway wall thickness and enhancing their propensity to constrict, leading to further airflow obstruction [40]. Eosinophils thicken the basement membrane, diminishing the airways’ ability to expand and recoil, which traps air in the lungs and worsens respiratory symptoms [4]. Additionally, persistent eosinophilic inflammation causes mucous glands to enlarge and produce more mucus, which blocks the airways and contributes to the chronic cough characteristic of COPD [41]. These intertwined processes clearly demonstrate how eosinophilic inflammation may exacerbate the structural degradation and functional decline in COPD.

Clinical research underscores the significant effects of eosinophilic inflammation on the progression of COPD [42]. Elevated eosinophil levels in blood and sputum correlate with a heightened risk of exacerbations—critical events that can accelerate disease progression, leading to further decline in lung function and increased disease severity [43,44]. These exacerbations are particularly damaging because when activated, eosinophils release a variety of inflammatory mediators such as eosinophil peroxidase, major basic protein, and eosinophil cationic protein [45].

The eosinophils produced cytokines implicated in emphysema pathogenesis, affect extracellular matrix production and fibrosis, contributing to lung structural changes [46]. Additionally, eosinophils generate reactive oxygen species (ROS) during inflammatory responses, adding to oxidative stress that further damages lung tissue and exacerbates COPD progression. Eosinophil-derived chemokines also attract other inflammatory cells like neutrophils and macrophages, whose enzymes and reactive species intensify emphysema symptoms and progression [47]. Furthermore, the presence of eosinophils influences the effectiveness of treatments in COPD [48,49] . This response underscores the potential benefits of focusing on eosinophilic pathways to alter the course of COPD, especially for patients with this inflammatory profile, suggesting that a personalized approach focusing on eosinophilic involvement could meaningfully alter the course of the disease.

## 6. Assessment of eosinophilic inflammation in COPD

Diagnosing eosinophilic COPD can be achieved through various methods. Fractional exhaled Nitric Oxide (FeNO) is an established biomarker that indicates underlying respiratory tract inflammation [50]. It offers a simple, non-invasive, and reproducible means to detect airway inflammation mediated by IL-13 and IL-4, and has been suggested to guide inhaled corticosteroid (ICS) use among asthma patients. However, FeNO is considered a less reliable marker of airway eosinophilia in COPD. Direct measurement of eosinophils in induced sputum provides a dependable indicator of eosinophilic airway inflammation and is regarded as the ‘gold standard’ for identifying airway inflammatory phenotypes, effectively predicting the risk of exacerbations [51]. Nevertheless, sputum induction is a semi-invasive procedure that requires specialized equipment and trained personnel. Bronchoalveolar lavage (BAL) provides a detailed profile of airway inflammation, including eosinophil count, making it useful in research settings [52]. However, limited studies were available on the clinical setting, particularly in eosinophilic COPD. Additionally, this invasive procedure involves bronchoscopy, which carries risks and discomfort for patients, making it unsuitable for regular monitoring due to its invasiveness and cost. Blood eosinophil count is a simple, widely available, and minimally invasive test that correlates reasonably well with sputum eosinophils and can predict the response to corticosteroids in COPD patients [44]. Peripheral eosinophilia is defined when the blood eosinophil count exceeds 0.5 × 10^9^/L (500/μL). Hence, the GOLD guidelines recommend using blood eosinophil counts as a biomarker to predict the efficacy of ICS therapy in COPD patients [2]. However, blood eosinophil levels can vary due to factors such as infections, medications, and comorbid conditions, which can affect their reliability as a biomarker. Additionally, a significant diurnal variability of blood eosinophil count has been described, with peak levels recorded around midnight and the lowest levels at midday [54]. A within-subject biological variation in hourly eosinophil count has also been noted. Higher variability in baseline eosinophil counts, defined as the difference between minimal and maximal eosinophil counts in a stable state, is associated with an increased risk of COPD exacerbations [54]. This highlights the importance of considering the variability of blood eosinophils when assessing exacerbation risk in COPD patients. Diagnostic strategies should consider the variability in eosinophil levels and preference of non-invasive methods to optimize treatment outcomes in eosinophilic COPD which can help define disease severity at presentation and guide treatment protocols.

## 7. Risk factors for eosinophilic inflammation in COPD

COPD often manifests with neutrophilic inflammation as a hallmark, but a notable subset of patients exhibits eosinophilic inflammation [55], which plays a significant role in influencing the disease’s progression, treatment strategies, and overall management. This variation in inflammatory response within the COPD population highlights the critical need for personalized treatments that can specifically target the underlying inflammatory mechanisms active in each individual.

Genetic predispositions play a key role in determining endotyping of eosinophilic inflammation in COPD [56], influencing disease severity and response to treatments such as corticosteroids [57,58]. Studies have pinpointed genes involved in eosinophil regulation and cytokine signaling, particularly those affecting IL-5 and IL-13 production, which are essential for eosinophil growth and activation [59,60].

Environmental factors, including air pollutants such as particulate matter, nitrogen dioxide, and ozone, also play a significant role in triggering eosinophilic inflammation [61,62]. Epidemiological studies have linked exposure to these pollutants with increased COPD exacerbations, indicating that chronic exposure may skew inflammation towards an eosinophilic phenotype, especially in genetically susceptible individuals [55, 63]. Furthermore, allergens trigger Th2 immune responses, leading to eosinophil recruitment and activation in the lungs, thus exacerbating COPD symptoms and increasing exacerbation risks [64].

COPD traditionally exhibits neutrophilic inflammation due to irritants like cigarette smoke, but some COPD patients, especially those experiencing exacerbations, also show elevated eosinophil counts. This dual-pathway inflammation contributes to the complexity and severity of the disease. Additionally, atopy and allergic rhinitis increase the risk of eosinophilic inflammation in COPD [65]. Individuals with these conditions, prone to allergic responses, might extend these eosinophilic reactions to the lungs, affecting COPD progression [66]. These insights into the common risk factors for eosinophilic inflammation in COPD. Understanding these dynamics allows for a more nuanced approach to COPD, ensuring that treatment strategies are as personalized and effective as possible.

## 8. Eosinophilic and non-eosinophilic inflammation contributing to COPD

In non-eosinophilic COPD, neutrophil host defense mechanisms appear to be compromised, with increased neutrophil migration, degranulation, and reactive oxygen species production in patients [74]. In contrast, in eosinophilic COPD, there are inflammatory signals that attract eosinophils to the lungs, where they release chemokines, cytokines, and cytotoxic granular products that contribute to inflammation. This eosinophilic response may enhance host defenses in allergic diseases, increasing susceptibility to exacerbations. Non-eosinophilic COPD patients exhibit higher sputum neutrophils, more frequent yearly exacerbations, increased co-morbidities, and greater bacterial burden in sputum cultures compared to eosinophilic patients [67]. Airway inflammation also plays a crucial role in host defense, and proper degradation of this inflammation is essential to maintaining homeostasis [68]. This process involves active molecular and cellular mechanisms that restore inflamed tissue to a stable state. Eosinophils are key players in modulating immune responses and inflammatory processes through lipid signaling pathways. In eosinophilic COPD, levels of mediators derived from 12/15-lipoxygenase (12/15-LOX) are significantly elevated compared to non-eosinophilic COPD [68]. One such mediator, 17-HDOHE, produced by 12/15-LOX, has been shown to enhance antibody-mediated immune responses, promote macrophage phagocytosis, and reduce inflammation [68]. This suggests that eosinophil recruitment in eosinophilic COPD may also facilitate the resolution of acute inflammation *via* a 12/15-LOX-initiated biosynthetic pathway. However, the outcomes of eosinophilic COPD compared to non-eosinophilic COPD still require further investigation and description.

## 9. Influence of eosinophilic inflammation on COPD management

The influence of eosinophilic inflammation on COPD management has garnered significant attention, challenging the traditional view that COPD is solely associated with neutrophil-driven inflammation from tobacco smoke exposure. Detection of elevated eosinophil levels in COPD, assessed through blood tests, is linked to distinct clinical characteristics, including an increased risk of exacerbations and a more rapid decline in lung function [69]. The presence of eosinophils in individuals with COPD is not merely a marker of inflammation but also plays a pivotal role in shaping the disease’s trajectory and response to therapies. An examination of data from clinical trials suggests that eosinophil counts could serve as a biomarker for adjusting treatment plans, particularly the application of inhaled corticosteroids (ICS), to mitigate exacerbation rates [70,71]. There is evidence for a reduced exacerbation rate in patients treated with ICS who have higher eosinophil levels, with cutoffs typically set at ≥2%, ≥150 cells/µL, or ≥300 cells/µL (Table 1) [53, 71–79]. Additionally, for instance, Watz et al. (2016) found a higher exacerbation rate when ICS was withdrawn from ICS-treated COPD patients with high eosinophil counts, supporting the beneficial effects of ICS for these patients [73]. Furthermore, a previous meta-analysis indicated that ICS reduced exacerbation rate according to the blood eosinophil count, with an inhibition rate of 20% at the ≥2% blood eosinophil threshold, 35% at ≥150 cells/µL, and 39% at ≥300 cells/µL [80]. The GOLD guidelines recommend that those COPD patients with a blood eosinophil count of ≥300 cells/µL as those most likely to benefit from ICS treatment [52]. In a previous RCT examining systemic corticosteroid treatment guided by blood eosinophil levels with a cutoff of <2% and ≥2%, it was found that among current smokers, the exacerbation rate was similar for both high and low eosinophil counts (11% vs. 12%, respectively). In contrast, among ex-smokers, the exacerbation rate was higher in those with high eosinophil counts compared to those with low eosinophil counts (48% vs. 22%, respectively). Thus, smoking status may have predictive value in systemic corticosteroid treatment in eosinophilic COPD. Moreover, patients with low eosinophil counts were reported to have a longer median hospital stay due to exacerbation compared to those with high eosinophil counts [81]. However, there was no difference in the time to re-hospitalization or time to death between the eosinophil strata. Another study indicated that patients with eosinophilic exacerbations had a shorter mean length of stay after treatment with oral corticosteroids, independent of prior treatment, compared to those with non-eosinophilic exacerbations [82]. Additionally, readmission rates at 12 months were similar between the groups. This finding underscores the nuanced role of eosinophils in COPD, which extends to influencing the disease’s progression, the frequency of exacerbations, and the efficacy of therapeutic interventions.

**Table 1.** Post-hoc analyses of randomized controlled trials with ICS-LABA or ICS-LABA-LAMA treatments in COPD based on blood eosinophil count.

Sources	Study design	Study arms	Eosinophil count subgroup	ICS response
Pascoe et al. (2015) [71]	double-blind RCT	Fluticasone + vilanterol (all doses) vs. vilanterol	<2% and ≥2%; <150 cells/μL and ≥150 cells/μL	Mean exacerbation rate (per year): Fluticasone + Vilanterol vs. Vilanterol Alone < 2% eosinophil: 0.79 vs. 0.89 ≥ 2% eosinophil: 0.91 vs. 1.28
Barnes et al. (2016) [72]	Double-blind RCT	Fluticasone vs. placebo	<2% and ≥2%	Mean exacerbation rate (per year): Fluticasone vs. placebo < 2% eosinophil: 1.32 vs. 1.63 ≥ 2% eosinophil: 1.59 vs. 1.81
Watz et al. (2016) [73]	Double-blind RCT	Fluticasone + tiotropium + salmeterol vs. tiotropium + salmeterol	<2% and ≥2%; <150 cells/μL and ≥150 cells/μL; <300 cells/μL and ≥300 cells/μL	Mean exacerbation rate (per year): Fluticasone + tiotropium + salmeterol vs. tiotropium + salmeterol < 2% eosinophil: 0.18 vs. 0.19 ≥ 2% eosinophil: 0.18 vs. 0.22
Papi et al. (2017) [74]	Double-blind RCT	Fluticasone + formoterol vs. formoterol	<2% and ≥2%	Mean exacerbation rate (per year): Fluticasone + formoterol vs. formoterol < 2% eosinophil: 0.70 vs. 0.84 ≥ 2% eosinophil: 0.88 vs. 0.88
Vestbo et al. (2017) [75]	Double-blind RCT	Beclometasone + formoterol + glycopyrronium vs. tiotropium	<2% and ≥2%	Adjusted rate ratio of exacerbation for Beclometasone + formoterol + glycopyrronium vs. tiotropium < 2% eosinophil: 0.933 ≥ 2% eosinophil: 0.700
Roche et al. (2017) [76]	Double-blind RCT	Fluticasone + salmeterol vs. indacaterol + glycopyrronium	<2% and ≥2%; <300 cells/μL and ≥300 cells/μL	Mean exacerbation rate (per year): Fluticasone + salmeterol vs. indacaterol + glycopyrronium < 2% eosinophil: 1.24 vs. 0.99 ≥ 2% eosinophil: 1.15 vs. 0.98
Chapman et al. (2018) [77]	Double-blind RCT	indacaterol + glycopyrronium vs. Fluticasone + tiotropium + salmeterol	<2% and ≥2%; <300 cells/μL and ≥300 cells/μL	Rate ratio of exacerbation for indacaterol + glycopyrronium vs. Fluticasone + tiotropium + salmeterol <300 cells/μL : 0.97 ≥ 300 cells/μL: 1.86
Papi et al. (2018) [78]	Double-blind RCT	Beclometasone/formoterol/ glycopyrronium vs. indacaterol/ glycopyrronium	<2% and ≥2%	Adjusted rate ratio of exacerbation for Beclometasone/formoterol/ glycopyrronium vs. indacaterol/ glycopyrronium < 2% eosinophil: 0.943 ≥ 2% eosinophil: 0.806
Ferguson et al. (2018) [79]	Double-blind RCT	Budesonide/glycopyrolate/ formoterol vs. glycopyrolate/ formoterol	<150 cells/μL and ≥150 cells/μL	Rate ratio of exacerbation for Budesonide/glycopyrolate/ formoterol vs. glycopyrolate/ formoterol < 150 cells/μL: 0.61 ≥ 150 cells/µL: 0.39
Lipson et al. (2018) [53]	Double-blind RCT	Fluticasone furoate/ umeclidinium/ vilanterol vs. umeclidinium/vilanterol	< 150 cells/μL and ≥150 cells/μL	Mean exacerbation rate (per year): Fluticasone furoate/ umeclidinium/ vilanterol vs. umeclidinium/vilanterol < 150 cells/μL: 1.06 vs. 0.97 ≥ 150 cells/µL: 0.95 vs. 1.39

RCT: randomized controlled trial; ICS: inhaled corticosteroid.

Additionally, biologic therapies targeting eosinophils, such as anti-IL-5 and anti-IL-33 monoclonal antibodies proven in eosinophilic COPD patients [83,84]. A Cochrane Review of anti-IL-5 (Mepolizumab) and anti-IL-5R (Benralizumab) in the treatment of COPD, demonstrating some evidence of potential benefits in select subgroups but not broad efficacy [60]. Anti-ST2 (Astegolimab) improved moderate-to-very severe COPD health status but did not significantly reduce exacerbation rate [85]. Anti-IL-33 (Itepekimab) has been shown to significantly reduce hospitalizations or emergency department visits in former smokers with moderate-to-severe COPD [86]. A summary of the randomized controlled trials investigating the use of anti-IL-4/IL-13 and anti-IL-5 therapies in COPD is presented in Table 2. In the treatment of eosinophilic COPD with anti-IL-5 therapy Mepolizumab, a significant reduction in mean blood and sputum eosinophil counts was observed, alongside an improvement in post-bronchodilator FEV1% compared to the placebo group [87]. The mean exacerbation rate in patients treated with Mepolizumab was reduced to 1.40 events per year, compared to 1.71 events per year in the placebo group [88]. The anti-IL-5 therapy Benralizumab demonstrated a numerically lower acute exacerbation rate by Poisson regression compared to placebo (0.39 vs. 0.76, respectively) in patients with eosinophil counts of ≥300 cells/µL [89]. The relative risk of exacerbations in patients with eosinophil counts ≥220 cells/µL was 0.69 when treated with Benralizumab 100 mg [90]. Additionally, the anti-IL-4/IL-13 therapy Dupilumab showed significant efficacy, with two studies reporting a reduction in exacerbation rates to 0.78 and 0.86 events per year, compared to 1.10 and 1.30 events per year in the placebo groups, respectively, in patients with eosinophil counts ≥300 cells/µL [91,92]. Anti-IL4Ra (Dupilumab) has been shown to reduce exacerbations, better lung function and quality of life, and less severe respiratory symptoms than the placebo group in COPD with eosinophilia [91]. Importantly, although not all of the METREX, METREO, GALATHEA, and TERRANOVA studies show statistically significant results, these studies provide insights into the effects of anti-IL-5 treatment in both eosinophilic and non-eosinophilic COPD [88, 90]. Conversely, the BOREAS and NOTUS studies, which investigated anti-IL-4/IL-13 therapies, enrolled COPD patients with a blood eosinophil count of 300 cells per microliter or higher to receive subcutaneous Dupilumab or a placebo biweekly [91,92]. The results demonstrated that adding Dupilumab to background triple inhaler therapy in patients with COPD and type 2 inflammation significantly decreased the annual rate of moderate or severe exacerbations and improved lung function [92]. On the other hand, there are still ongoing trials investigating the effects of monoclonal antibodies on eosinophilic COPD, including MATINEE (mepolizumab), RESOLUTE (benralizumab), and COURSE (tezepelumab). In an abstract presentation, it was reported that the Phase IIa COURSE trial demonstrated a significant 37% reduction in the rate of moderate or severe exacerbations compared to placebo in patients with blood eosinophil counts ≥150 cells/µL treated with tezepelumab [93]. Additionally, in patients with blood eosinophil counts ≥300 cells/µL, there was a reduction of 46% in the rate of moderate or severe exacerbations [93]. These developments have been facilitated by elucidating key molecular pathways involved in eosinophilic inflammation, paving the way for targeted therapeutic strategies that promise to enhance patient outcomes in COPD. This approach not only allows for treatments to be tailored to the inflammatory profiles of individual patients but also minimizes potential side effects associated with systemic corticosteroid therapies.

**Table 2.** Randomized controlled trials with anti-IL-5 or anti-IL5Ra or anti-IL4Ra antibody treatments in COPD.

Monoclonal antibody	Sources	Study design	Study arms	Eosinophil count subgroup	Duration of treatment	Treatment response	
						Intervention	Placebo
Anti-IL-5	Dasgupta et al. (2017) [87]	Double-blind RCT	mepolizumab vs. placebo	–	3 months and 6 months	- Mean blood eosinophil: 0.04 cells/mm^3^ (3 months) and 0.03 cells/mm^3^ (6 months) - Mean sputum eosinophil: 0.75% (3 months) and 0.5% (6 months) - Mean FEV1% post bronchodilator: 65.50% (3 months) and 63.50% (6 months)	- Mean blood eosinophil: 0.23 cells/mm^3^ (3 months) and 0.26 cells/mm^3^ (6 months) - Mean sputum eosinophil: 3.15% (3 months) and 2.20% (6 months) - Mean FEV1% post bronchodilator: 43.50% (3 months) and 43.50% (6 months)
Anti-IL-5	Pavord et al. (2017) [88]	Double-blind RCT	mepolizumab vs. placebo	<150 cells/µL, ≥150 cells/µL or ≥300 cells/µL	12 months	Mean exacerbation rate in eosinophilic COPD: 1.40 events/year	Mean exacerbation rate in eosinophilic COPD: 1.71 events/year
Anti-IL-5	Brightling et al. (2014) [89]	Double-blind RCT	Benralizumab vs. placebo	<200 cells/µL, ≥200 cells/μL or ≥300 cells/μL	12 months	- Mean acute exacerbation rate by Poisson regression: 0.39 in ≥300 cells/µL - Mean pre-bronchodilator FEV1 change: 0.13 L	- Mean acute exacerbation rate by Poisson regression: 0.76 in ≥300 cells/µL - Mean pre-bronchodilator FEV1 change: −0·06 L
Anti-IL-5	Criner et al. (2019) [90]	Double-blind RCT	Benralizumab vs. placebo	<220 cells/µL or ≥220 cells/μL	12 months	Relative risk (RR) of exacerbation in ≥220 cells/μL with benralizumab 100 mg: 0.88 (95% CI 0·77–0·99)	
Anti-IL-4/IL-13	Bhatt et al. (2023) [91]	Double-blind RCT	Dupilumab vs. placebo	≥300 cells/μL	12 months	Mean exacerbation rate: 0.78 events/year	Mean exacerbation rate: 1.10 events/year
Anti-IL-4/IL-13	Bhatt et al. (2024) [92]	Double-blind RCT	Dupilumab vs. placebo	≥300 cells/μL	12 months	Mean exacerbation rate: 0.86 events/year	Mean exacerbation rate: 1.30 events/year

RCT: randomized controlled trial.

The adoption of eosinophil counts as a biomarker has ushered in a stratified approach to COPD treatment, embodying the principles of precision medicine. This approach tailors treatments based on individual characteristics, such as eosinophil levels, promoting personalized treatment plans [6, 42, 69]. Evolving guidelines now suggest adjusting ICS doses based on eosinophil thresholds, reflecting a move towards more customized care [7, 80]. Research into COPD cohorts reveals that a notable proportion of patients consistently exhibit elevated eosinophil levels over extended periods [32]. These insights highlight the potential of eosinophil count monitoring as a tool for identifying patients who may benefit from tailored therapeutic strategies, including interventions specifically targeting eosinophilic inflammation. This shift towards acknowledging the heterogeneity of COPD and the need for tailored treatments based on individual inflammatory profiles marks a significant advancement in the management of the disease.

However, the clinical management of asthma and COPD also exhibits distinct differences, particularly in the approach and intensity of ICS use and the strategies for exacerbation prevention. Asthma management generally involves an early and more aggressive use of ICS combined with long-acting beta agonists (LABA), focusing on long-term control and trigger avoidance [94]. In contrast, COPD management emphasizes conservative ICS use, prioritizes bronchodilators as the foundation of therapy, and incorporates broader strategies like smoking cessation and comorbidity management to address the disease’s progressive nature and associated health issues [95]. These differences underscore the need for tailored treatment protocols that reflect the unique aspects of each condition. The eosinophilic phenotype of COPD presents a unique challenge and opportunity for precision medicine. Identifying this phenotype allows for a more nuanced approach to treatment, with strategies tailored to address the specific inflammatory processes at play. A previous publication has reported a decrease in eosinophils after ICS treatment, with the pharmacological target of ICS believed to be that of type 2 inflammation [96]. COPD patients with elevated blood and lung eosinophil counts show increased T2-associated gene expression, which correlated with higher response to ICS treatment [97]**.** Further analysis identified distinct subgroups of COPD patients with varying transcriptome signatures related to T2 inflammation, inflammasome activation, or mitochondrial activation; however, only the T2 signature was suppressed following ICS intervention [96,97]. Therefore, these observations indicate that ICS may suppress eosinophils in COPD patients through targeting T2-related inflammation. In COPD patients with eosinophil-associated airway inflammation, ICS treatment resulted in a 62% reduction in severe exacerbations leading to hospitalization [69, 98]. The combination of the ICS, fluticasone furoate, with the long-acting beta-agonist, vilanterol, significantly reduced the rate of moderate/severe exacerbations compared to vilanterol alone in patients with eosinophil counts ≥ 2% [69, 71]. These findings underscore the potential utility of blood eosinophil counts not only as diagnostic biomarker but as a predictor of ICS treatment response. Furthermore, patients with persistent eosinophilia tend to exhibit milder COPD symptoms and a more gradual progression of emphysema, suggesting a nuanced role of eosinophils in the disease’s natural history [35]. The Withdrawal of Inhaled Steroids During Optimized Bronchodilator Management (WISDOM) study indicated that while ICS can be safely withdrawn in some COPD patients, those with elevated eosinophil levels may benefit from continued ICS therapy [99]. The European Respiratory Society has issued guidelines recommending ICS withdrawal in patients with low exacerbation risk, particularly those with eosinophil counts below 300 cells/μL [100]. For patients with higher eosinophil counts (≥300 cells/μL), continued ICS therapy is advised due to their higher exacerbation risk and potential better response to ICS [100]. These guidelines also strongly recommend using one or two long-acting bronchodilators if ICS is withdrawn [100]. These points underscore the critical role of eosinophil counts in informing the decision to initiate ICS treatment in eosinophilic COPD. These findings underscore the potential utility of eosinophil counts not just as diagnostic biomarkers but as predictors of disease trajectory and treatment response.

## 10. Charting the future: exploring new horizons in eosinophilic COPD research

The promise of personalized medicine in managing eosinophilic COPD is on the horizon, necessitating research into how best to integrate individual patient data, including biomarker profiles, into clinical decision-making processes. Additionally, the development of novel therapeutic agents targeting eosinophilic pathways offers a tantalizing prospect for more effective treatments, meriting rigorous clinical evaluation to ascertain their safety, efficacy, and long-term benefits.

In conclusion, the journey through the landscape of eosinophilic COPD is one of discovery and innovation, with the promise of improved patient outcomes and more personalized care on the horizon. The continued collaboration of researchers, clinicians, and patients will be pivotal in turning the potential of current insights into tangible benefits for those living with this complex and challenging disease.

## Data Availability

Data sharing is not applicable to this article as no new data were created or analyzed in this study.
